# Bifurcation percutaneous coronary intervention of left main trunk treated using a proximal optimizing technique and proximal balloon edge dilation technique

**DOI:** 10.1016/j.jccase.2021.06.002

**Published:** 2021-07-03

**Authors:** Teruyoshi Kume, Satoshi Koto, Yasuyuki Sudo, Okamoto Hiroshi, Ryotaro Yamada, Koichiro Imai, Terumasa Koyama, Tomoko Tamada, Yoji Neishi, Shiro Uemura

**Affiliations:** Department of Cardiology, Kawasaki Medical School, Kurashiki, Japan

**Keywords:** Optical coherence tomography, Bifurcation, Percutaneous coronary intervention

## Abstract

We present a case of bifurcation percutaneous coronary intervention (PCI) of the left main trunk (LMT) using a proximal balloon edge dilation (PBED) technique following a proximal optimizing technique (POT). The procedure of the PBED technique entailed precise positioning of the balloon for SB dilation, with the proximal radiopaque marker lying in the cross-sectional plane of the stent struts at the left circumflex artery (LCx) ostium. The PBED technique might prevent stent deformation induced by side branch (SB) dilation and eliminates the need for the second POT procedure in the re-POT sequence. In fact, three-dimensional reconstruction of optical coherence tomography (3D-OCT) revealed good opening of stent cells overlying the LCx ostium without deformation of stent struts causing incomplete stent apposition at the site opposite the LCx, so the second POT procedure was unnecessary in this case.

<**Learning objective:** This is the first case report to describe bifurcation PCI of the LMT using POT-PBED procedures under 3D-OCT guidance. POT-PBED procedures might offer excellent acute results for cross-over single-stent implantation in LMT bifurcation lesions and could eliminate the second POT procedure in the re-POT sequence.>

## Introduction

The default approach in bifurcation percutaneous coronary intervention (PCI) generally involves simple strategies with a single stent or provisional side branch (SB) stenting [1]. We previously reported an experimental bifurcation bench model study that showed good effects on obstruction by stent struts at a jailed SB ostium using a proximal balloon edge dilation (PBED) technique in the repetitive proximal optimizing technique (re-POT) sequence compared with conventional SB balloon dilation[2]. SB dilation with the PBED technique prevented stent deformation induced by SB dilation and eliminated the need for the second POT procedure in the re-POT sequence. In this case report, we performed PCI in a left main trunk (LMT) bifurcation lesion using PBED technique and the three-dimensional reconstruction (3D) of optical coherence tomography (OCT) (Abbott Vascular, Santa Clara, CA) showed excellent acute results after the PCI procedure.

## Case report

**Fig. 1:**
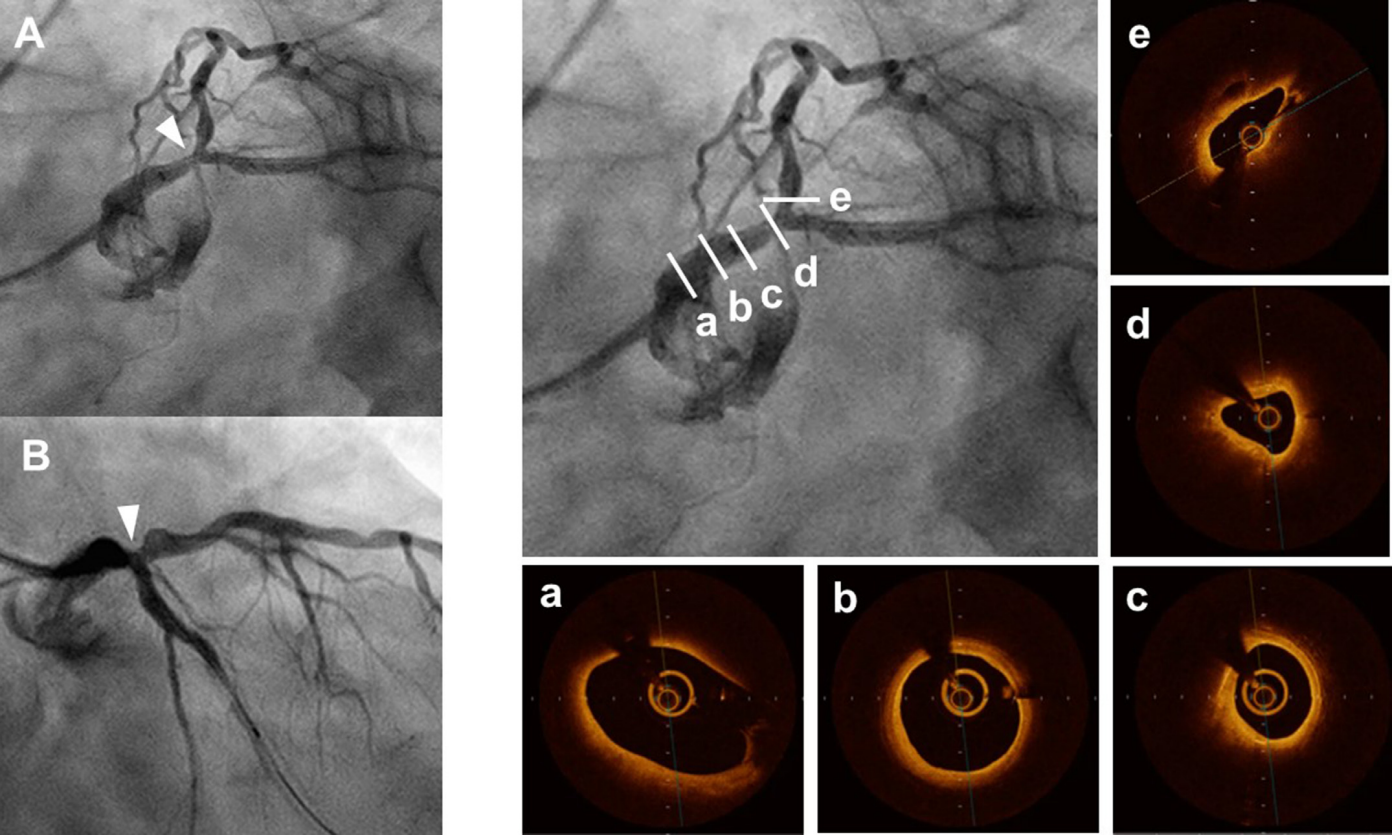
Coronary angiogram (A, B) and optical coherence tomography (a-e) before the procedure. Left anterior oblique caudal view (A) and right anterior oblique caudal view (B) of the left coronary artery show 75% stenosis at the distal left main trunk (arrowhead). Optical coherence tomography (OCT) successfully visualizes the proximal left main trunk (LMT) through the Telescope™ guide extension catheter (a-c). OCT evaluation of distal LMT lesion reveals a 4.1-mm^2^ minimal luminal area in a 23-mm long lesion showing a thick cap-fibroatheroma plaque with calcification (d).

**Fig. 2:**
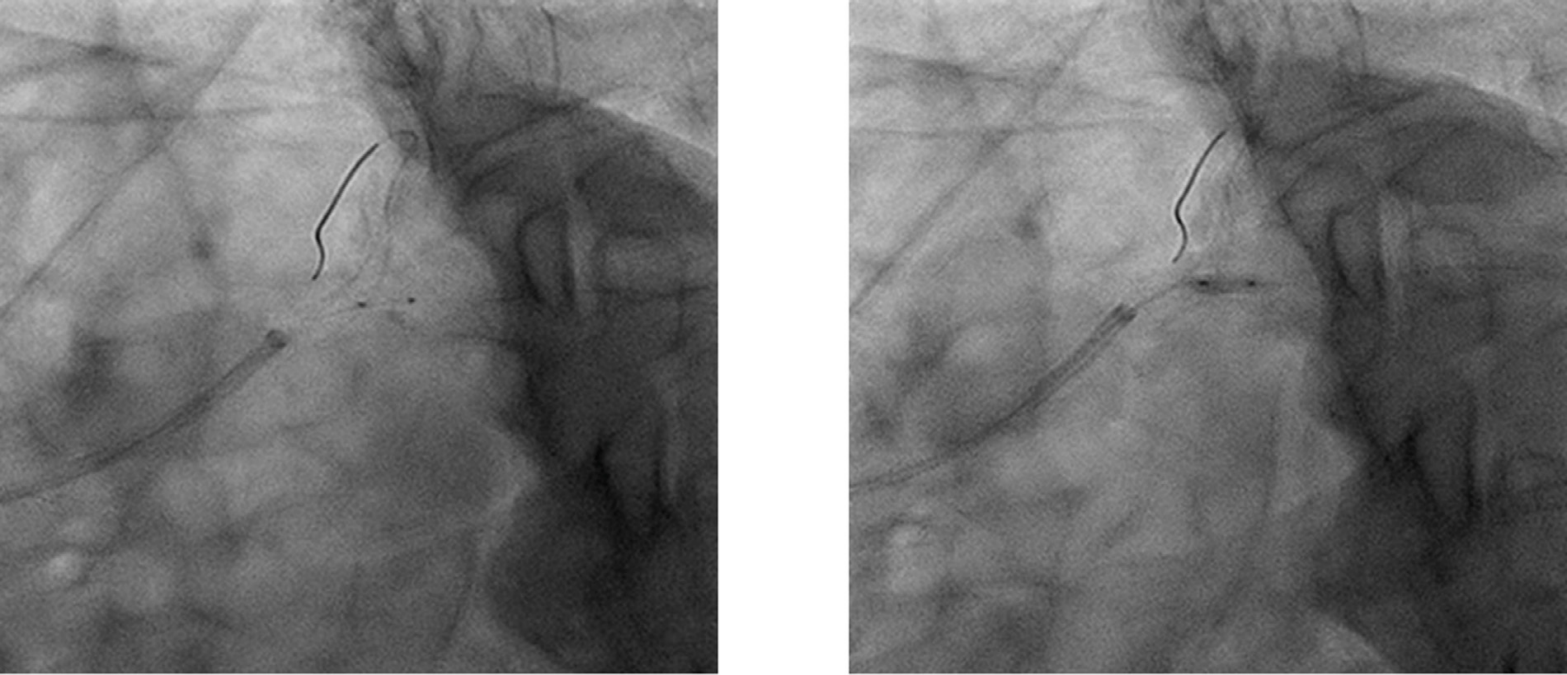
Coronary angiogram during side branch dilatation using a proximal balloon edge dilation (PBED) technique. Side branch (left circumflex artery: LCx) dilatation with a 2.5 mm × 6 mm non-compliant balloon is performed using the PBED technique. The procedure for the PBED technique involves precise positioning of the balloon for side branch dilation, with the proximal radiopaque marker lying in the cross-sectional plane of the stent struts at the LCx ostium (left).

**Fig. 3:**
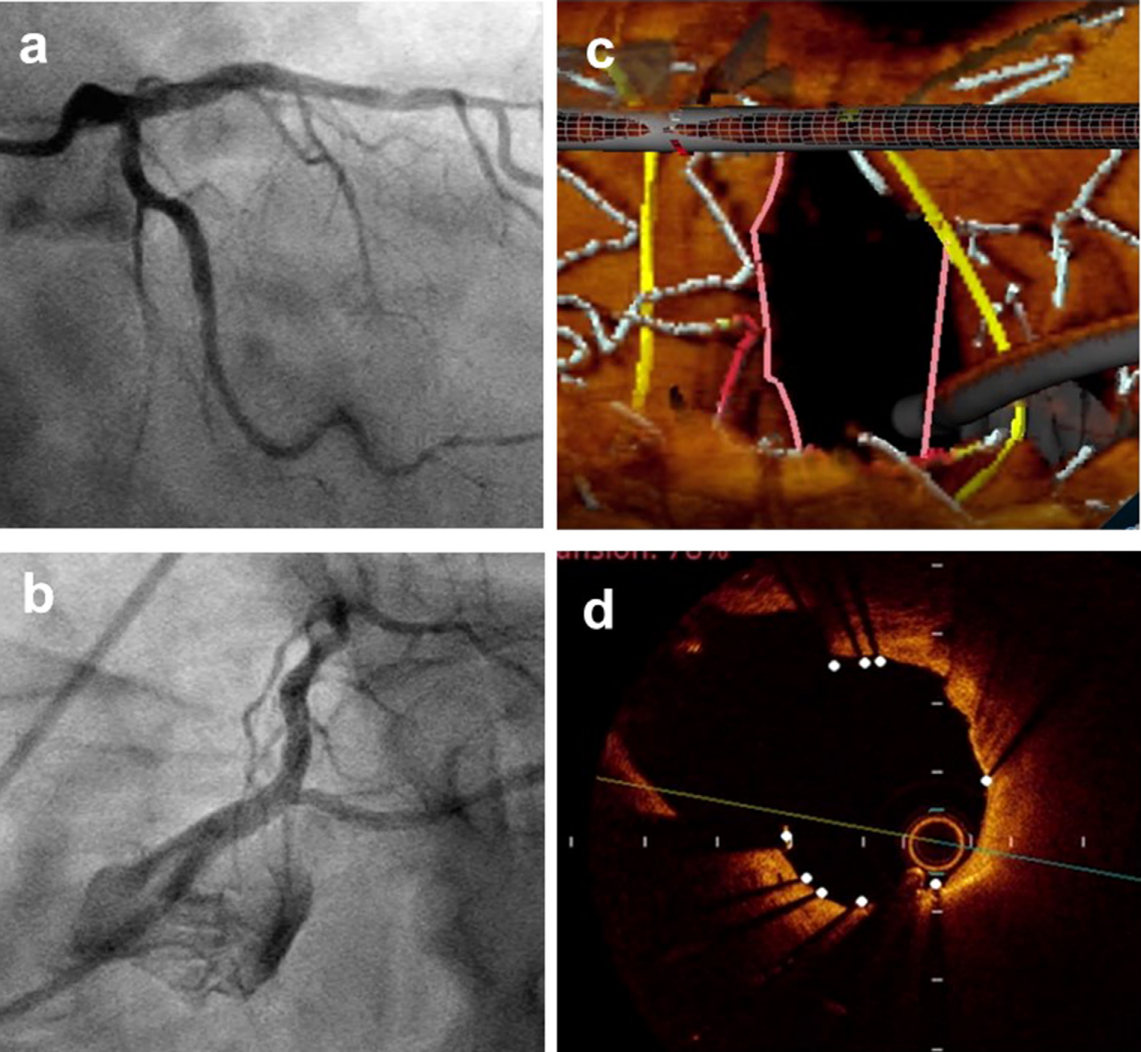
Coronary angiogram (CAG: a, b) and optical coherence tomography (OCT: c, d) after the procedure. After successful side branch (SB) dilatation using the proximal balloon edge dilation (PBED) technique, CAG shows good angiographic results (a, b) and three-dimensional reconstruction of OCT reveals good opening of stent cells overlying the SB ostium (c). OCT demonstrates well-apposed stent struts opposite the SB ostium (d).

A 67-year-old man with acute coronary syndrome was transferred to our hospital. Emergency coronary angiography (CAG) showed total occlusion of the mid-right coronary artery and primary PCI was performed. One month after successful primary PCI, physiological assessment by fractional flow reserve detected residual ischemia at the distal LMT. We thus decided to perform bifurcation PCI (Medina's classification 1,1,0) under 3D-OCT guidance. CAG showed 75% stenosis at the distal LMT (Fig. 1, A and B). Using a Telescope™ guide extension catheter (Medtronic Cardiovascular, Santa Rosa, CA), OCT evaluation of the distal LMT lesion revealed a 4.1-mm^2^ minimal luminal area of a 23-mm long lesion having a thick cap-fibroatheroma plaque with calcification (Fig. 1, a-e). Proximal and distal reference vessel diameters were 4.5 mm and 3.5 mm, respectively, and we implanted a 3.5 mm × 23 mm everolimus-eluting stent (Xience Skypoint™ Abbott Vascular, Santa Clara, CA) from the mid-LMT to the proximal left anterior descending artery lesion, crossing over the left circumflex artery (LCx). After stent implantation, a proximal optimization technique (POT) was performed with a 4.5 mm × 8 mm non-compliant balloon to fully appose the proximal part of the stent in the LMT. After rewiring to the LCx, no links connecting to a carina and appropriate re-crossing positions of the wire were confirmed using 3D-OCT. SB (LCx) dilation with a 2.5 mm × 6 mm non-compliant balloon using the PBED technique was performed. The procedure for the PBED technique was that the balloon for SB dilation was positioned precisely, with the proximal radiopaque marker lying in the cross-sectional plane of the stent struts at the LCx ostium (Fig. 2). After successful SB dilatation using the PBED technique, CAG showed good angiographic results (Fig. 3, a, b) and 3D-OCT revealed good opening of stent cells overlying the SB (LCx) ostium (Fig. 3, c). Lumen area at the ostium of LCX did not change during the POT-PBED procedures (from 4.5mm^2^ to 4.4 mm^2^). OCT demonstrated well-apposed stent struts opposite the LCx (Fig. 3, d, arrowhead). We thus decided not to perform the second POT (re-POT) procedure because incomplete stent apposition was not observed at either the proximal stent edge of the LMT or the side opposite the LCx on OCT.

## Discussion

An informative experimental study demonstrated that a re-POT sequence, comprising initial POT, SB inflation, and second POT, significantly improved the final results of cross-over single-stent implantation for bifurcation lesions [3], and these good results were confirmed in a multicenter clinical trial [4]. However, we have previously reported that the second POT procedure in the re-POT sequence increased obstruction of stent struts at a jailed SB ostium, because deformation of stent cells at the main branch occurred during SB inflation to open the SB ostium in our experimental bifurcation bench model study [5]. More recently, to prevent stent deformation during SB dilatation, our experimental bifurcation bench model study introduced the utility of SB inflation using the PBED technique and showed improved final results of cross-over single-stent implantation in bifurcation lesions using the POT-PBED procedures [2]. In the present case, OCT demonstrated well-apposed stent struts at both the proximal stent edge of the LMT and opposite the LCx after POT-PBED procedures, and a second POT (re-POT) procedure was not needed. POT-PBED procedures could eliminate the second POT procedure in the re-POT sequence, and thus reduce procedure time and avoid stent deformation due to insertion of the second POT balloon. Very short balloon (length 4 mm), such as Glider™ balloon (TriReme Medical, Pleasanton, CA, USA) or ZINRAI balloon (Kaneka Medix Corporation, Osaka, Japan) could bear similar effects of PBED technique that avoid stent deformation during SB dilatation. These very short balloons might be recommended for SB dilatation in bifurcation lesions treated with cross-over single-stent implantation as well.

Experimental comparison data between the POT-PBED procedures and kissing balloon technique that is widely used in real practice are not available. Future experimental investigations are necessary to reveal the advantage of PBED technique against kissing balloon technique.

In conclusion, POT-PBED procedures showed excellent acute results for cross-over single-stent implantation in an LMT bifurcation lesion visualized by 3D-OCT.

## Disclose

All other authors have nothing to disclose.
